# Essentials of Medical Electricity

**Published:** 1892-09

**Authors:** 


ESSENTIALS OF MEDICAL ELECTRICITY.—By D. D. Stewart, M. D., and E. S. Lawrence, M. D., Philadelphia. W. B. Saunders, 913 Walnut St., Philadelphia.

THE first line of preface states, "the writers of this manual lay no claim to originality." The competitive labor to present to the student, "a general idea of the diagnostic and therapeutic value of electricity." In this they have succeeded, and to those who are interested in the subject, this monogram will prove of value. A small book, neatly and substantially bound, contains 158 pages. There are a number of cuts, illustrations of instruments, and therapeutic application of electricity. The price is $1.00. The authors are experienced teachers in the Jefferson Medical College. A. B. P.

